# Leptin Is Associated with Poor Clinical Outcomes and Promotes Clear Cell Renal Cell Carcinoma Progression

**DOI:** 10.3390/biom11030431

**Published:** 2021-03-15

**Authors:** Wen-Lang Fan, Yuan-Ming Yeh, Tsung-Ta Liu, Wei-Ming Lin, Tse-Yen Yang, Chao-Wei Lee, Tsung-Chieh Lin

**Affiliations:** 1 Genomic Medicine Core Laboratory, Chang Gung Memorial Hospital, Linkou 33305, Taiwan; alangfan@gmail.com (W.-L.F.); Yeh234@gmail.com (Y.-M.Y.); 2 Division of Gastroenterology, Tri-Service General Hospital, Taipei 11490, Taiwan; darling9968@pchome.com.tw; 3 Department of Diagnostic Radiology, Chang Gung Memorial Hospital, Chiayi Branch, Chang Gung University of Science and Technology, Chiayi 61363, Taiwan; weiming276@gmail.com; 4 Center of Augmented Intelligence in Healthcare, China Medical University Hospital, Taichung 40447, Taiwan; hardawayoung@gmail.com; 5 Molecular and Genomic Epidemiology Center, Department of Medical Research, China Medical University Hospital, Taichung 40447, Taiwan; 6 Center for General Education, Master Program of Digital Health Innovation, College of Humanities and Sciences, China Medical University, Taichung 40447, Taiwan; 7 Department of Biotechnology, College of Medical and Health Science, Asia University, Taichung 41354, Taiwan; 8 Division of General Surgery, Department of Surgery, Linkou Chang Gung Memorial Hospital, Taoyuan 33305, Taiwan; alanchaoweilee@hotmail.com; 9 Graduate Institute of Clinical Medical Sciences, Chang Gung University, Taoyuan 33305, Taiwan; 10 College of Medicine, Chang Gung University, Taoyuan 33302, Taiwan

**Keywords:** leptin, renal cell carcinoma, clinical outcome

## Abstract

Emerging evidence has shown the oncogenic roles of leptin in modulating cancer progression in addition to its original roles. Analyses of transcriptomic data and patients’ clinical information have revealed leptin’s prognostic significance in renal cell carcinoma (RCC). However, its biological effects on RCC progression have not yet been explored. Clinical and transcriptomic data of a RCC cohort of 603 patients were retrieved from The Cancer Genome Atlas (TCGA) and analyzed to reveal the correlation of leptin with clinical outcomes and the hierarchical clustering of gene signatures based on leptin levels. In addition, cox univariate and multivariate regression analyses, cell migration upon leptin treatment, identification of putative leptin-regulated canonical pathways via ingenuity pathway analysis (IPA), and the investigation of induction of Wnt5a, ROR2, and Jun N-terminal Kinases (JNK) phosphorylation activation were performed. We first observed a correlation of high leptin levels and poor outcomes in RCC patients. Knowledge-based analysis by IPA indicated the induction of cancer cell migration by leptin, which was manifested via direct leptin treatment in the RCC cell lines. In RCC patients with high leptin levels, the planar cell polarity (PCP)/JNK signaling pathway was shown to be activated, and genes in the axis, including CTHRC1, FZD2, FZD10, ROR2, WNT2, WNT4, WNT10B, WNT5A, WNT5B, and WNT7B, were upregulated. All of these genes were associated with unfavorable clinical outcomes. WNT5A and ROR2 are pivotal upstream regulators of PCP/JNK signaling, and their correlations with leptin expression levels were displayed by a Pearson correlation analysis. The inhibition of signal transduction by SP600125 reversed leptin-mediated cell migration properties in RCC cell lines. The results indicate the prognostic impact of leptin on RCC patients and uncover its ability to promote cell migration via PCP/JNK signaling.

## 1. Introduction

Emerging evidence has demonstrated leptin’s roles in regulating cancer progression, including tumor proliferation, metastasis, angiogenesis, and drug resistance [1]. Leptin is the product of the *Ob* (*LEP*) gene, which was cloned in 1994 by Friedman and colleagues. It was noted that the gene was also called leptin after the Greek “leptos”, meaning thin [2]. Leptin was previously characterized as a peptide hormone secreted by adipocytes, and it functions as the ligand of leptin receptor (*LEPR*), which regulates energy expenditure and hunger [3,4]. Recent findings have shown leptin and leptin receptor expression beyond traditional tissues, thereby suggesting the signaling pathway’s pivotal role outside of its physiological modulation. Several tissues express leptin, including the placenta, stomach, fibroblasts, mammary epithelium, and skeletal muscle [5,6,7,8]. Furthermore, data from public databases indicate leptin RNA expression in a broad range of cancer types, including kidney cancer. Renal cell carcinoma (RCC) is a malignancy derived from renal tubular epithelial cells and is a common malignant tumor in the urinary system; its incidence rate is second to the bladder cancer [9]. Approximately 85% of RCC patients are diagnosed as subtype of RCC, clear cell RCC (ccRCC) [9]. ccRCC is insensitive to traditional radiotherapy or chemotherapy, and the most common therapeutic strategy is nephrectomy [10]. However, approximately 20 to 30% of patients have distant metastases at the time of diagnosis, and around 30% of patients in the aforementioned cohort progress to local recurrence or distant metastasis after nephrectomy for localized disease [11,12]. Critical molecular alterations in ccRCC include the von Hippel–Lindau (VHL) gene mutation, VHL promoter hypermethylation, and chromosome 3p deletion which support the distinct pathogenesis [13]. However, leptin’s effects on regulating tumor progression as well as its prognostic significance in ccRCC have not yet been addressed. In this study, the clinical role of leptin in RCC focused on its prognostic value in predicting survival rate. In addition, the potential biological function was pre-analyzed by knowledge-based software according to the transcriptomic data in cancer patients. The leptin-mediated impacts on ccRCC progression were investigated as well as addressing the molecular mechanism. We aimed to shed light on the critical role of leptin in ccRCC and provide evidence for developing a therapeutic strategy for those patients displaying high leptin levels.

## 2. Results

### 2.1. Leptin Is an Independent Predictor of a High Hazard Ratio for ccRCC, and the Hierarchical Clustering of Transcriptomic Data Is Shown Based on High and Low Leptin Groups in the ccRCC Cohort

**Figure 1:**
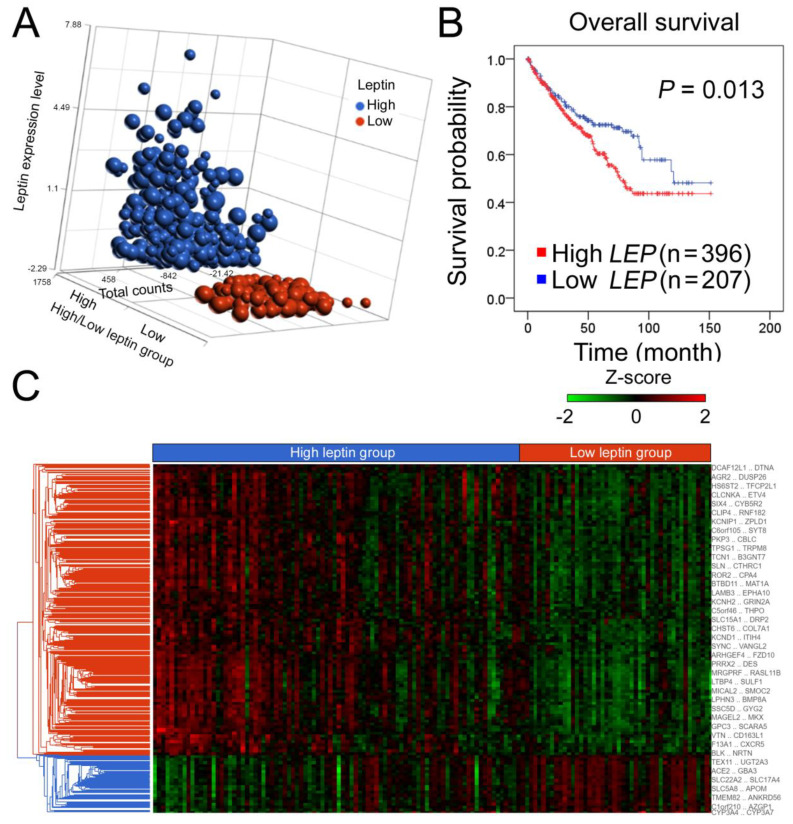
Correlation of leptin with clinical outcomes and hierarchical clustering of gene signatures based on leptin levels. The gene expression profile in the dataset of kidney renal clear cell carcinoma (KIRC) (dataset ID: Table 2. PANCAN) was determined by RNA-Seq platform (Illumina HiSeq). (**A**) Relative leptin and transcriptomic read counts (total counts) of clear cell renal cell carcinoma (ccRCC) patients in the leptin high/low groups; 603 cases were stratified to 396 cases in high-leptin group and 207 cases in low-leptin group. Size of dot indicates the relative case number of patients. (**B**) Kaplan–Meier analysis of leptin expression under the condition of overall survival probability in ccRCC patients. (**C**) Hierarchical clustering of genes in the high and low leptin groups. Z-scores were computed on a gene-by-gene basis by subtracting the mean and then dividing by the standard deviation.

We first explored leptin’s clinical significance in ccRCC. Clinical information and transcriptomic data of a ccRCC cohort of 603 patients were retrieved from The Cancer Genome Atlas (TCGA) and investigated [14]. Patients were stratified into high and low groups based on the relative LEP expression levels (Figure 1A and Supplementary Table S1). High leptin expression was associated with poor overall survival (*p* = 0.013, Figure 1B). Furthermore, the results of the univariate Cox regression analysis indicated that high leptin expression levels were correlated with poor outcomes. Leptin further appeared to serve as an independent predictor for high hazard ratios compared with the patient’s tumor (T), node (N), and metastasis (M) (TNM) status and clinical stage (*p* = 0.006, multivariate, Table 1). These data indicate the prognostic application of leptin for ccRCC patients. An analysis of hierarchical clustering in high/low leptin groups displayed a roughly distinguished pattern, suggesting the merit of investigating transcriptomic changes to justify the aforementioned clinical observations (Figure 1C).

### 2.2. Knowledge-Based Transcriptomic Analysis Reveals Leptin’s Potential Effect in Promoting Cancer Metastasis

**Figure 2:**
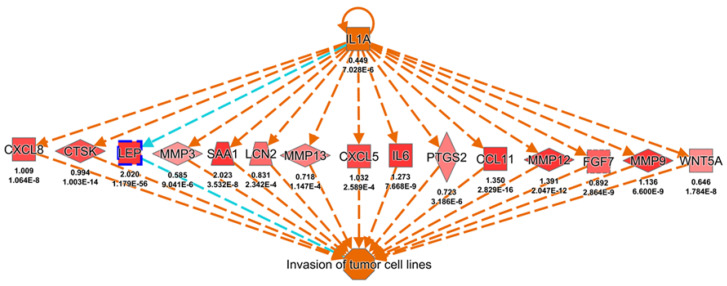
Transcriptomic alteration-based identification of potential diseases and functions modulated upon leptin upregulation. A total of 20,530 gene expression data from each ccRCC patient were retrieved from The Cancer Genome Atlas TCGA. The differential gene signatures were obtained after comparing each gene in the high leptin group to each gene in the low leptin group. Gene targets were further filtered by the log2-transformed 1.5-fold change and *p* value (<0.05) for ingenuity pathway analysis (IPA) (938 gene targets). The putative regulatory network of the top diseases and functions is presented. E: e notation indicated “× 10*^n^*” of a value *m* × 10*^n^*.

To further explore the potential phenotypes that might be regulated upon leptin upregulation leading to poor clinical outcomes in cancer patients, those RNA-Seq data from the ccRCC cohort from the TCGA were investigated (Supplementary Table S2). A total of 938 gene targets were identified as significant differential expression in the high leptin group when compared with the low leptin group, and were selected for further study via the Regulator Effects module in the knowledge-based ingenuity pathway analysis (IPA) platform. The potential regulation of leptin toward cancer cell migration and invasion was identified and listed (top three diseases and functions, Table 2). The one with the top consistency score is shown, and it is also noted that leptin might promote tumor cell invasion (Figure 2).

### 2.3. Leptin Promotes ccRCC Migration

**Figure 3:**
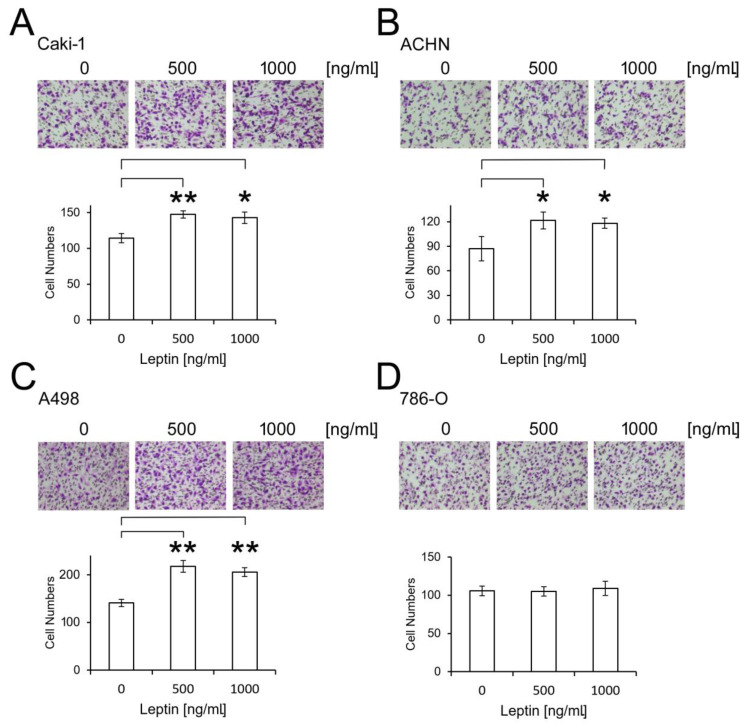
Investigation of ccRCC cell migration upon leptin treatment. Leptin (500 and 1000 ng/mL) was added to the ccRCC cell lines Caki-1 (**A**), ACHN (**B**), A498 (**C**), and 786-O (**D**) for 24 h. Cancer cell migration ability was then evaluated by transwell assays. The experiments were performed three times and a representative result is shown. The *p* values at the following levels were considered significant: * *p* < 0.05, ** *p* < 0.01.

Cancer cell migration ability was then examined by transwell assays. ccRCC cell lines were treated with leptin at concentrations of 500 or 1000 ng/mL prior to performing the assay. The treatments with dosages lower than 500 ng/mL revealed the nonsignificant effects in RCC cell lines. Caki-1, ACHN, and A498 cells exhibited a significant increase in cell migration upon leptin addition, though the effect was not observed in 786-O cells (*p* < 0.05–0.01, Figure 3). These data further indicate leptin’s regulatory effect in inducing ccRCC migration, which might contribute to cancer progression, especially in patients with leptin overexpression.

### 2.4. The Planar Cell Polarity (PCP) Signaling Pathway Is Predictively Activated by Leptin

**Figure 4:**
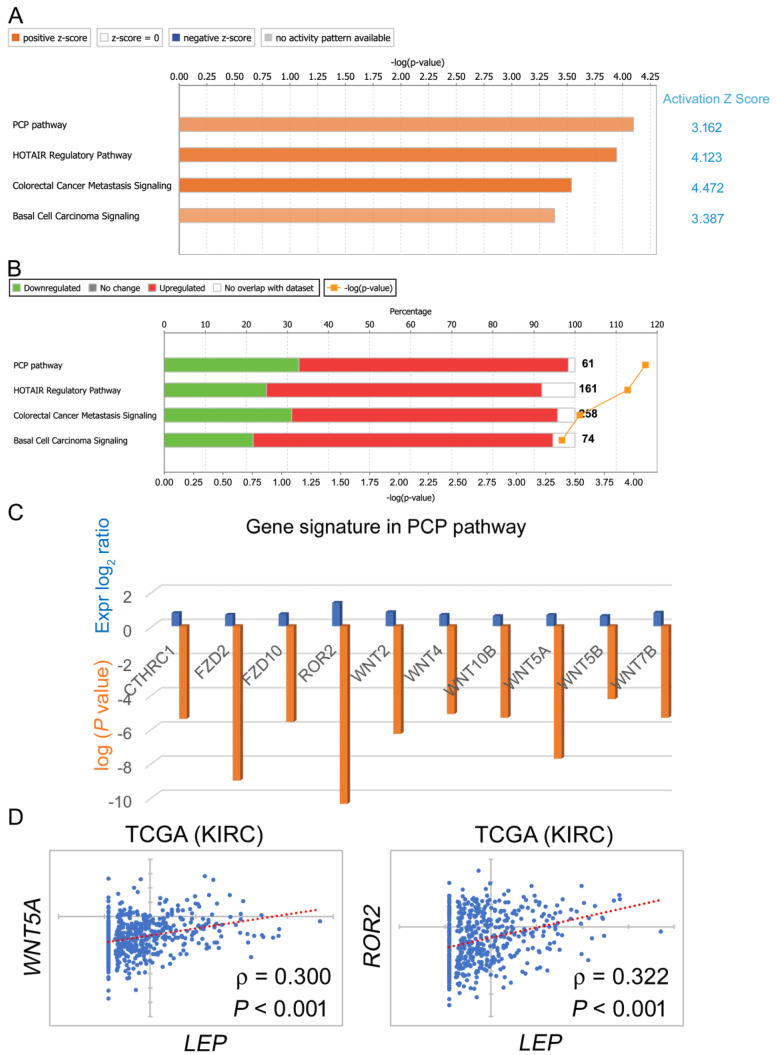
List of putative leptin-regulated canonical pathways via IPA. (**A**) The differentially expressed gene targets in the high leptin group were studied in the IPA platform. Pivotal canonical pathways judged by the levels of gene expression and numbers of target overlapping with the IPA database are shown along with the activation status judged by the z-score. (**B**) The distribution of up- and downregulated gene targets in each canonical pathway, (**C**) gene targets characterized with the significant expressional alterations in the PCP signaling pathway, and (**D**) Pearson correlations of WNT5A and ROR2 with LEP in the TCGA ccRCC cohort.

We next aimed to study the molecular mechanism in leptin-dependent ccRCC migration. High-throughput screening-based gene signature alterations in the high leptin group were examined via IPA. Critical canonical pathways were characterized based on the level of gene target overlap as well as the corresponding expression pattern with the IPA database. Among those predicted signaling pathways, the PCP signaling axis was the top pathway judged by significant *p* value and activation status (activation z-score = 3.162, Figure 4A). PCP signaling is one of the noncanonical Wnt signaling pathways of whose aberrant activation could lead to tumor migratory properties [15,16,17,18]. The distribution of up- and downregulated gene targets is shown in Figure 4B. Ten gene targets of the PCP pathway, namely, CTHRC1, FZD2, FZD10, ROR2, WNT2, WNT4, WNT10B, WNT5A, WNT5B, and WNT7B, were further identified in the high leptin group (Figure 4C). Importantly, WNT5A and ROR2 act upstream of the PCP signaling pathway [19,20]. A significant correlation of both WNT5A and ROR2 (*p* < 0.001, Figure 4D) as well as another eight genes (*p* < 0.001, Figure S2) with LEP were observed in the ccRCC cohort, indicating the potential regulatory function of leptin in activating the PCP axis.

### 2.5. Gene Targets in the PCP Pathway Reveal Their Prognostic Significance in ccRCC

**Figure 5:**
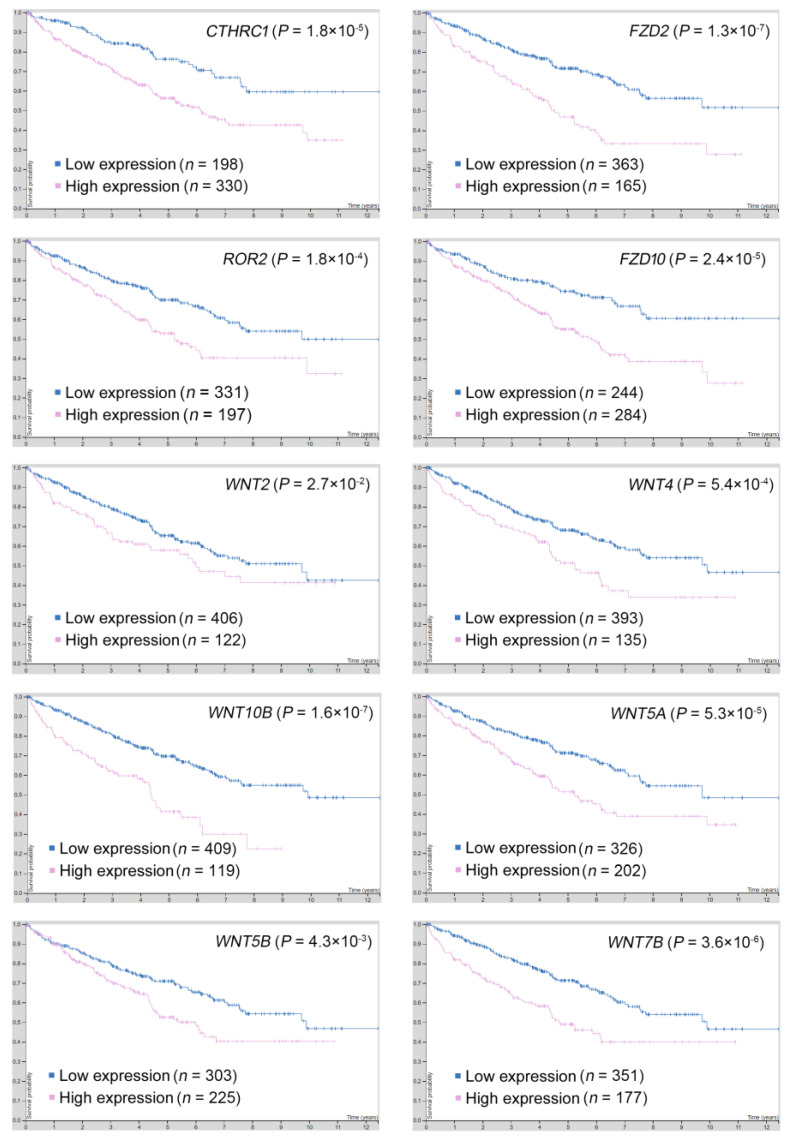
Prognostic significance of PCP pathway gene targets in the ccRCC cohort. The prognostic significance in predicting overall survival was shown after stratifying RNA levels of the indicated genes in the ccRCC cohort (TCGA). The clinical and transcriptomic data of ccRCC patients were obtained from The Pathology Atlas database (https://www.proteinatlas.org/; accessed on 15 January 2021).

Ten gene targets in the PCP pathway were further studied to examine their clinical significance in ccRCC (TCGA). The expression profiles and clinical follow-up data were investigated and retrieved in the Human Protein Atlas/The Pathology Atlas database [21,22,23,24]. Importantly, CTHRC1, FZD2, FZD10, ROR2, WNT2, WNT4, WNT10B, WNT5A, WNT5B, and WNT7B all showed an association with poor overall survival and were upregulated in the high leptin group (Figure 5). These results first highlight the pivotal prognostic role of the PCP pathway in ccRCC patients and the value of further dissecting the corresponding molecular mechanisms of cancer progression.

### 2.6. Leptin Triggers Cancer Cell Migration via the PCP/JNK Signaling Pathway

**Figure 6:**
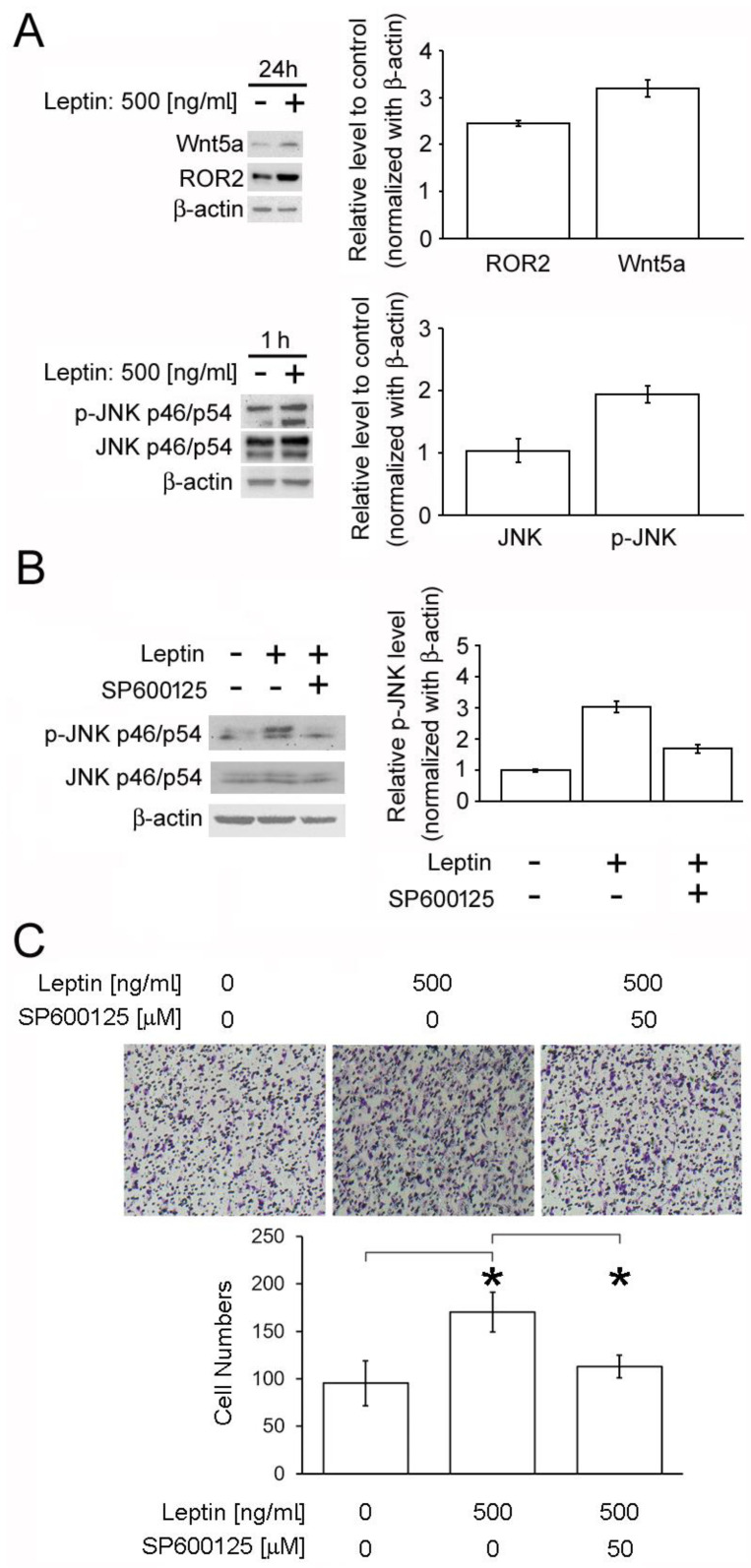
Induction of JNK phosphorylation activation and cell migration by leptin. (**A**) ACHN cells were treated with 500 ng/mL leptin for 1 and 24 h. The p-JNK at p46 and p54 isoforms and Wnt5a and ROR2 levels were detected by Western blot. (**B**) ACHN cells were pretreated with 50 μM SP600125 (a JNK inhibitor) for 30 min prior to 1 h of leptin incubation. Relative p-JNK level was evaluated by Western blot. (**C**) ACHN cells were pretreated with 50 μM SP600125 (a JNK inhibitor) for 30 min prior to 24 h of leptin incubation. Cell migration ability was measured by transwell assays. The experiments were performed three times and a representative result is shown. The *p* values at the following levels were considered significant: * *p* < 0.05.

The noncanonical WNT/PCP pathway is known to play a pivotal role in promoting cell movement associated with JNK phosphorylation activation [20,25,26]. An immunoblot was performed on ACHN cells to test for the level of JNK and JNK phosphorylation. The results showed the upregulation Wnt5a and a slight alternation of ROR2 in ACHN cells after leptin treatment (upper panel, Figure 6A). In addition, ROR2, WNT5A, WNT5B, WNT4, FZD10, and FZD2 expression were also observed to be upregulated by 12 h of leptin treatment (Figure S3), suggesting the positive regulatory role of leptin in activating the PCP signaling pathway. Simultaneously, a significant phosphorylation activation of JNK upon leptin addition at an early stage was detected (lower panel, Figure 6A). Therefore, the consequence of JNK inhibition was then studied. The block of the signal transduction via the JNK inhibitor SP600125 appeared to reverse the leptin-mediated increase of JNK activation and cell migration in ACHN cells (Figure 6B,C). Similar phenotype was observed in A498 and Cak-1 cells (Figure S1). These data suggest the pivotal role of leptin/PCP/JNK signaling activation in ccRCC progression.

## 3. Discussion

The clear cell subtype of RCC displays relatively high level of leptin receptor [1]. 786-O had also been pathologically characterized as a clear cell type with stable *VHL* mutation [27], which might be similar to other three clear cell subtype cell lines studied in this manuscript. However, according to the public data released by the Cancer Cell Line Encyclopedia (CCLE) database [24,25], 786-O cells revealed the lower expression of leptin receptor (*LEPR*) than other cell lines which might in part explain the difference upon the same dose of leptin treatment (Table S3). In addition, the discrepancy observed in 786-O cells might be simultaneous due to the insensitivity of leptin receptor to leptin stimulus. However, the detailed molecular mechanism remains to be further explored.

Results from recent publications further focused on secreted leptin regarding to its role in RCC carcinogenesis and progression. Leptin was higher in the conditioned media of human adipose explants from kidney cancer tissue, and the incubation with RCC cell lines appeared to decrease cancer cell adhesion and increase cell migration [26]. However, several meta-analysis studies demonstrated that serum leptin might not sufficiently reflect the risk and progression of RCC and serve as a biomarker for early detection [27,28,29]. It is noted that leptin is a peptide hormone whose level in serum is closely related to energy expenditure which may be one of difficulties in addition to the control of leptin stability during clinical measurement.

Leptin-mediated regulation toward JNK signaling is reported in several types of cancer. In ovarian cancer, leptin was found to induce matrix metalloproteinase 7 expression to promote cell invasion by activating ERK and JNK pathways [28]. In addition, leptin was found to increase cell proliferation in MCF-7 breast cancer cells via aromatase activation and JNK phosphorylation [29]. Furthermore, leptin promoted colorectal cancer cell growth through the metallopanstimulin-1 (MPS-1)-dependent activation of the JNK signaling pathway [30]. In Barrett’s esophageal adenocarcinoma, leptin appeared to increase cell proliferation and abolish apoptosis via the transactivation of the epidermal growth factor receptor and JNK activation [31]. To date, JNK activation by leptin has not been addressed in RCC. In this study, we showed its regulatory effect toward RCC migration.

Increasing clinical studies indicate the correlation of leptin with cancer metastasis [1]. The correlation of the expression of leptin and its receptor with bone metastasis has been reported in pulmonary adenocarcinoma patients [32]. Cutaneous melanoma patients who have relatively high serum leptin expression levels are at a significant risk of sentinel lymph node metastasis [33]. Furthermore, the results of a correlation analysis in an endometrial cancer cohort demonstrated the positive association of lymph node metastasis with high leptin and leptin receptor levels [34]. In addition to the phenotype of increased cancer cell migration observed in this study, leptin has been reported to regulate metastasis in various types of cancer. Leptin induces cell migration, invasion, and metastasis in an orthotopic model of pancreatic cancer, and the simultaneous increase in the expression of leptin receptor and MMP13 also shows a positive association with the TNM stage [35].

The clinical implications and underlying mechanisms linking excess adiposity and cancer promotion development and metastasis have been identified and reported [36]. In addition, the activation of noncanonical Wnt signaling through WNT5A in visceral adipose tissue of obese subjects is related to inflammation which is similar to the consequence caused upon leptin treatment [37]. Since endogenous control genes in human adipose tissue were identified and the relevance to obesity and obesity-associated type 2 diabetes mellitus was found [38], it would be interesting to know if the patients classified in the high leptin group were also those with a higher BMI. However, BMI data of patients were not collected and included in clear cell type of RCC (ccRCC) datasets from TCGA. Hence, the correlation remains to be further explored.

## 4. Materials and Methods

### 4.1. TCGA Dataset

A renal clear cell carcinoma cohort was studied. The clinical information of the cohort including age at initial diagnosis, gender, pathological TNM status, targeted molecular therapy, smoking history, and additional pharmaceutical/radiation therapy are integrated in Table S4. A total of 603 cases were stratified to 396 cases in high leptin group and 207 cases in low leptin group judged by leptin expression level and coordinate overall survival. Gene expression in a kidney renal clear cell carcinoma (KIRC) dataset of TCGA (dataset ID: TCGA_KIRC_exp_HiSeqV2_PANCAN) was previously determined by RNA-Seq analysis (Illumina HiSeq). Data were retrieved for further in-house analysis. RNA read counts were normalized and log2 transformed.

### 4.2. Ingenuity Pathway Analysis (IPA)

Ingenuity^®^ Pathway Analysis (QIAGEN, Hilden, Germany; www.qiagen.com/ingenuity; accessed on 15 October 2020) was performed according to the pipeline which was previous described [39]. Briefly, differential gene expression signatures of the ccRCC cohort stratified to high and low leptin groups were uploaded and analyzed by the Ingenuity^®^ Pathway Analysis platform according to the instructions.

### 4.3. Cell Culture

All human renal adenocarcinoma cell lines were obtained from the American Type Culture Collection (Manassas, VA, USA) and were gifts from Dr. Michael Hsiao of the Genomics Research Center at Academia Sinica in Taiwan. Culture methods and experimental conditions were according to our previous publication [39].

### 4.4. Cell Migration Assay

The method followed the precedent research article [39]. In vitro migration was assessed by transwell assays with membrane pore size of 8 μm (Millipore, Bedford, MA, USA). A total of 2 × 10^5^ cells in serum-free culture medium were studied in each transwell experiment. A498, ACHN, and Caki-1 cells were allowed to migrate for 3.5 h, and migration time for 786-O cells was 0.5 h.

### 4.5. Western Blot Analysis

The method and experimental conditions were previously described [39]. After blocking with 5% nonfat milk, the membrane was incubated with specific primary antibodies (p-JNK: sc-6254, Santa Cruz, Dallas, TX, USA, 1:1000; β-actin: sc-47778, Santa Cruz, Dallas, TX, USA, 1:10,000) overnight at 4 °C.

### 4.6. Statistical Analysis

All data are presented as mean ± S.D. The *p* values at the following levels were considered significant: * *p* < 0.05, ** *p* < 0.01, and *** *p* < 0.001. For estimates of the survival rates, clinical follow-up data were calculated by the Kaplan–Meier method, and were compared using the log-rank test. In addition, student’s *t*-test was applied for other statistical analyses.

## 5. Conclusions

The results indicate the prognostic significance of leptin in predicting the unfavored overall survival in RCC patients, and uncover its ability to promote cell migration of RCC cells via the activation of the PCP/JNK signaling pathway.

## Figures and Tables

**Table 1:** Cox univariate and multivariate regression analyses of the TNM prognostic factors, pathological stage, and leptin level for overall survival in 603 patients with renal cell carcinoma.

		Univariate		Multivariate	
Variable	Comparison	HR (95% CI)	*p*-Value	HR (95% CI)	*p*-Value
Sex	M:F	0.939 (0.704–1.253)	0.670	0.924 (0.607–1.404)	0.924
Stage	3-4:1-2	3.817 (2.784–5.233)	<0.001	1.733 (0.685–4.385)	0.246
T	T3-4:T1-2	3.138 (2.320–4.245)	<0.001	1.370 (0.604–3.110)	0.451
N	N1:N0	3.380 (1.795–6.367)	<0.001	1.677 (0.860–3.268)	0.129
M	M1:M0	4.486 (3.372–5.967)	<0.001	2.458 (1.482–4.079)	<0.001
Leptin	High:low	1.479 (1.084–2.018)	0.013	2.044 (1.224–3.416)	0.006

Cox proportional hazards regression analysis was applied to evaluate the independent prognostic contribution of leptin after accounting for other critical covariates. F, female; M, male; HR, hazard ratio; CI, confidence interval.

**Table 2:** Top 10 diseases and functions ranked by the consistency score.

ID	Consistency Score	Regulators	Diseases and Functions
1	3.615	IL1A	Invasion of tumor cell lines
2	3.5	IL17A	Invasion of cells
3	3.491	PI3K (complex)	Migration of cells
4	3.464	EGFR	Size of body
5	3.464	cyclic AMP	Activation of cells
6	3.411	PI3K (complex)	Cell movement
7	3.357	Cigarette smoke	Cell proliferation of tumor cell lines
8	3.333	CCL11	Migration of cells
9	3.317	Alpha catenin	Invasion of tumor cell lines
10	3.317	IL22	Activation of cells

## Data Availability

Not applicable.

## Supplementary Materials

The following are available online at https://www.mdpi.com/2218-273X/11/3/431/s1, Figure S1: Induction of cell migration via JNK phosphorylation by leptin, Figure S2: Pearson correlations of indicated molecular targets with *LEP* in the TCGA ccRCC cohort were evaluated, Figure S3: The expressions of indicated targets upon leptin treatment in ACHN cells, Table S1: Hierarchical Clustering, Table S2: IPA, Table S3: CCLE database, Table S4: Clinical information of TCGA cohort.
