# Supplementary material for: Leptin Is Associated with Poor Clinical Outcomes and Promotes Clear Cell Renal Cell Carcinoma Progression
Source: Biomolecules. 2021 Mar 15;11(3):431. doi: 10.3390/biom11030431 (PMC7999177; doi:10.3390/biom11030431)
Supplement: Supplementary file 1 [file biomolecules-11-00431-s001.zip › Supplementary Files/List of primers in Supplementary Figure 3.rtf]

WNT5A
Input PCR template
NM_003392.7 Homo sapiens Wnt family member 5A (WNT5A), transcript variant 1, mRNA 
Range 322 - 1464
Specificity of primers Primers may not be specific to the input PCR template as targets were found in selected database:Refseq mRNA (Organism limited to Homo sapiens)...help on specific primers

Primer pair 4
	Sequence (5'->3')	Template strand	Length	Start	Stop	Tm	GC%	Self complementarity	Self 3' complementarity	
Forward primer	CTCCTTCGCCCAGGTTGTAA	Plus	20	423	442	59.68	55.00	4.00	2.00	
Reverse primer	TGTCCTTGAGAAAGTCCTGCC	Minus	21	557	537	59.93	52.38	3.00	1.00	
Product length	135	


CTHRC1
Input PCR template
AY358914.1 Homo sapiens clone DNA76393 CTHRC1 (UNQ762) mRNA, complete cds 
Range 138 - 869
Specificity of primers Primer pairs are specific to input template as no other targets were found in selected database: Refseq mRNA (Organism limited to Homo sapiens)

Primer pair 4
	Sequence (5'->3')	Template strand	Length	Start	Stop	Tm	GC%	Self complementarity	Self 3' complementarity	
Forward primer	CCCTGGGGCCAATGTTATT	Plus	19	341	359	57.37	52.63	5.00	3.00	
Reverse primer	GCTTGTAGTTGGGTGTCCAG	Minus	20	459	440	58.48	55.00	3.00	1.00	
Product length	119	


FZD2
Input PCR template
NM_001466.4 Homo sapiens frizzled class receptor 2 (FZD2), mRNA 
Range 206 - 1903
Specificity of primers Primer pairs are specific to input template as no other targets were found in selected database: Refseq mRNA (Organism limited to Homo sapiens)

Primer pair 1
	Sequence (5'->3')	Template strand	Length	Start	Stop	Tm	GC%	Self complementarity	Self 3' complementarity	
Forward primer	CCGTGCCGCTCTATCTGTG	Plus	19	530	548	60.59	63.16	3.00	0.00	
Reverse primer	GTCCTCGGAGTGGTTCTGGC	Minus	20	679	660	62.51	65.00	5.00	2.00	
Product length	150	


FZD10
Input PCR template
AB027464.1 Homo sapiens FZD10 mRNA for Frizzled-10, complete cds 
Range 18 - 1763
Specificity of primers Primer pairs are specific to input template as no other targets were found in selected database: Refseq mRNA (Organism limited to Homo sapiens)

Primer pair 1
	Sequence (5'->3')	Template strand	Length	Start	Stop	Tm	GC%	Self complementarity	Self 3' complementarity	
Forward primer	GCTCAAGTGCTCCCCGATTA	Plus	20	359	378	59.82	55.00	2.00	2.00	
Reverse primer	GCCTCCATGCACAGGTAGTT	Minus	20	466	447	60.04	55.00	4.00	0.00	
Product length	108	


ROR2
Input PCR template
M97639.1 Human transmembrane receptor (ror2) mRNA, complete cds 
Range 200 - 3031
Specificity of primers Primers may not be specific to the input PCR template as targets were found in selected database:Refseq mRNA (Organism limited to Homo sapiens)...help on specific primers

Primer pair 4
	Sequence (5'->3')	Template strand	Length	Start	Stop	Tm	GC%	Self complementarity	Self 3' complementarity	
Forward primer	CACCAACGGGATGAAGACCA	Plus	20	610	629	59.96	55.00	2.00	0.00	
Reverse primer	GTAAGGCTGGCAGAACCCAT	Minus	20	730	711	60.03	55.00	5.00	2.00	
Product length	121	


WNT2
Input PCR template
NM_003391.3 Homo sapiens Wnt family member 2 (WNT2), transcript variant 1, mRNA 
Range 70 - 1152
Specificity of primers Primer pairs are specific to input template as no other targets were found in selected database: Refseq mRNA (Organism limited to Homo sapiens)

Primer pair 9
	Sequence (5'->3')	Template strand	Length	Start	Stop	Tm	GC%	Self complementarity	Self 3' complementarity	
Forward primer	CTCGGTGGAATCTGGCTCTG	Plus	20	82	101	60.18	60.00	3.00	1.00	
Reverse primer	GCACATTATCGCACATCACCC	Minus	21	202	182	60.00	52.38	3.00	0.00	
Product length	121	


WNT4
Input PCR template
AB061675.1 Homo sapiens mRNA for WNT4, complete cds 
Range 6 - 1061
Specificity of primers Primer pairs are specific to input template as no other targets were found in selected database: Refseq mRNA (Organism limited to Homo sapiens)

Primer pair 4
	Sequence (5'->3')	Template strand	Length	Start	Stop	Tm	GC%	Self complementarity	Self 3' complementarity	
Forward primer	GTCCCCGCTCGTGCC	Plus	15	10	24	59.75	80.00	4.00	2.00	
Reverse primer	GCCCTTGAGTTTCTCGCAC	Minus	19	149	131	59.13	57.89	3.00	0.00	
Product length	140	


WNT10B
Input PCR template
U81787.1 Human Wnt10B mRNA, complete cds 
Range 364 - 1533
Specificity of primers Primers may not be specific to the input PCR template as targets were found in selected database:Refseq mRNA (Organism limited to Homo sapiens)...help on specific primers

Primer pair 7
	Sequence (5'->3')	Template strand	Length	Start	Stop	Tm	GC%	Self complementarity	Self 3' complementarity	
Forward primer	CTTCAGGGTCTGCACATCGC	Plus	20	580	599	61.36	60.00	4.00	2.00	
Reverse primer	GAAACCGCGCTTGAGGATG	Minus	19	705	687	59.57	57.89	6.00	0.00	
Product length	126	


WNT5B
Input PCR template
AB060966.1 Homo sapiens mRNA for WNT5B, complete cds 
Range 6 - 1085
Specificity of primers Primers may not be specific to the input PCR template as targets were found in selected database:Refseq mRNA (Organism limited to Homo sapiens)...help on specific primers

Primer pair 1
	Sequence (5'->3')	Template strand	Length	Start	Stop	Tm	GC%	Self complementarity	Self 3' complementarity	
Forward primer	CAACCAGTTCAAGAGCGTGC	Plus	20	974	993	60.04	55.00	4.00	2.00	
Reverse primer	TACTGGTCCACGATCTCCGT	Minus	20	1072	1053	60.03	55.00	5.00	2.00	
Product length	99	


WNT7B
Input PCR template
AF416743.1 Homo sapiens WNT7B (WNT7B) mRNA, complete cds 
Range 375 - 1424
Specificity of primers Primer pairs are specific to input template as no other targets were found in selected database: Refseq mRNA (Organism limited to Homo sapiens)

Primer pair 4
	Sequence (5'->3')	Template strand	Length	Start	Stop	Tm	GC%	Self complementarity	Self 3' complementarity	
Forward primer	CTACGTGTTTCTCTGCTTTGGC	Plus	22	404	425	60.10	50.00	4.00	2.00	
Reverse primer	TTGCAGATGATGTTGGCTCCC	Minus	21	490	470	60.96	52.38	4.00	1.00	
Product length	87	
