# Supplementary material for: Leptin Is Associated with Poor Clinical Outcomes and Promotes Clear Cell Renal Cell Carcinoma Progression
Source: Biomolecules. 2021 Mar 15;11(3):431. doi: 10.3390/biom11030431 (PMC7999177; doi:10.3390/biom11030431)
Supplement: Supplementary file 1 [file biomolecules-11-00431-s001.zip › Supplementary Files/Supplementary Figure S1.rtf]

Supplementary Data


Supplementary Figure S1. Induction of cell migration via JNK phosphorylation by leptin. Caki-1 cells (A) and A498 Cells (B) were pretreated with 50 M SP600125 (a JNK inhibitor) for 30 min prior to 24 h of 500 ng/ml leptin incubation. Cell migration ability was measured by transwell assays.
