# Supplementary material for: Leptin Is Associated with Poor Clinical Outcomes and Promotes Clear Cell Renal Cell Carcinoma Progression
Source: Biomolecules. 2021 Mar 15;11(3):431. doi: 10.3390/biom11030431 (PMC7999177; doi:10.3390/biom11030431)
Supplement: Supplementary file 1 [file biomolecules-11-00431-s001.zip › Supplementary Files/Supplementary Figure S2.rtf]

Supplementary Figure S2. Pearson correlations of indicated molecular targets with LEP in the TCGA ccRCC cohort were evaluated.
