# Supplementary material for: Leptin Is Associated with Poor Clinical Outcomes and Promotes Clear Cell Renal Cell Carcinoma Progression
Source: Biomolecules. 2021 Mar 15;11(3):431. doi: 10.3390/biom11030431 (PMC7999177; doi:10.3390/biom11030431)
Supplement: Supplementary file 1 [file biomolecules-11-00431-s001.zip › Supplementary Files/Supplementary Figure S3.rtf]

Supplementary Figure S3. The expressions of indicated targets upon leptin treatment in ACHN cells. Relative expressional levels were measured by real-time PCR upon 12 h of leptin treatments.
